# 3D anatomical digital twins: New generation virtual models to navigate robotic partial nephrectomy

**DOI:** 10.1002/bco2.453

**Published:** 2025-02-17

**Authors:** Daniele Amparore, Alberto Piana, Federico Piramide, Sabrina De Cillis, Enrico Checcucci, Cristian Fiori, Francesco Porpiglia

**Affiliations:** ^1^ Department of Urology, San Luigi Gonzaga Hospital University of Turin Turin Italy; ^2^ Department of Surgery Candiolo Cancer Institute, FPO‐IRCCS Turin Italy

**Keywords:** image‐guided surgery, intraoperative‐guidance, nephron‐sparing surgery, planning, robotic surgery, three‐dimensional imaging

## Abstract

Objective 3D virtual models have gained interest in urology, particularly in the context of robotic partial nephrectomy. From these, newly developed “anatomical digital twin models” reproduce both the morphological and anatomical characteristics of the organs, including the texture of the tissues they comprise. The aim of the study was to develop and test the new digital twins in the setting of intraoperative guidance during robotic‐assisted partial nephrectomy (RAPN). Patient and Methods The production path of the 3D model‐digital twin of an organ begins with a phantom of virtual elements, including the kidney's parenchyma, vessels, tumour and collecting system. Textures are created from intraoperative robotic surgery images using machine learning algorithms. The result is a 3D model ‐ digital twin that replicates the organ's shape and appearance. Two surgeons, one experienced and one young, used both the standard 3D model and the digital twin in four surgical phases: identifying the organ and its boundaries, dissecting the vascular pedicle, isolating the neoplastic lesion and identifying the renal pelvis and ureter. Results 4 patients, 2 per each surgeon harbouring a low and intermediate complexity (PADUA 6 and 8) renal masses respectively, underwent RAPN. From the assessment made by the surgeons at the end of each procedure, the 3D digital twin models were found to be superior to their standard counterparts both in terms of concordance with real anatomy and in usefulness to guide the identification of the tumour, vascular pedicle and ureter, while they did not demonstrate significant advantages in identifying the kidney and its margins. Conclusions The new 3D digital twin models represent a step forward towards the personalization of virtual reconstructions. Approaching real anatomy more closely, they offer the surgeons a perceived higher degree of concordance with the intraoperative environment, making it easier to identify the structures of interest during the surgical procedure.

## 1 INTRODUCTION AND BACKGROUND

In recent years, 3D virtual models have gained interest in the field of urology, from preoperative planning to intraoperative guidance during surgical procedures, particularly in the context of robotic partial nephrectomy.^1^

The production process of these models has evolved in parallel with the development of technologies for reconstructing two‐dimensional images from CT scans and MRI, integrating various software tools and involving different professionals such as bioengineers, radiologists and surgeons.^2^

Several generations of reconstructions have been developed, leading to the definition of HA3D virtual models.^3^

As the generations of HA3D virtual models have progressed, the level of accuracy has gradually increased thanks to the introduction of mathematical algorithms integrated within the reconstruction software script which allow for precise calculation and representation of the perfusion volumes of the kidney,^4^ facilitating selective clamping planning during partial nephrectomy.^5^

Furthermore, in the context of conservative renal surgery, some anatomical details have been added to the virtual model to assist the surgeon in better planning renal parenchyma resection and suturing, representing intrarenal structures with chromatic variations based on their distance from the margin of the renal mass to be removed.

While these models faithfully replicate organ morphology, they lack the textural nuances of real organs. Thus, even if valuable for planning and understanding organ anatomy, especially for novices,^6^ they may not accurately represent real surgical conditions, as the plain colours visible on the digital model may not be observable looking at the organ textures in vivo.

Based on these needs, the concept of an “anatomical digital twin” for urological surgical planning was developed.

The “digital twin” is defined a virtual representation of a physical object, process or system that utilizes real‐time data to model, simulate and analyse its behaviour.^7^ It originated in the context of manufacturing and the Internet of Things (IoT), allowing for more efficient monitoring, optimization and management of real‐world objects. Essentially, it represents a digital version of something real to understand its functioning and continuously improve it.^8^

In this perspective, a digital model of urological organs has been developed, reproducing both their morphological and anatomical characteristics, thus including the texture of the tissues they comprise. This new model should enhance the surgeon's visual correspondence between the virtual organ and the real one during planning and intraoperative guidance.

## 2 AIM OF THE STUDY AND METHODS

The aim of this study was to develop and test the new digital twins in the setting of intraoperative guidance during robotic‐assisted partial nephrectomy (RAPN). One experienced and one young surgeon were selected for the study. They had the opportunity to consult both the standard 3D model and the 3D digital twin model during their surgeries, with both models displayed on the two screens of the robotic console using the Tile‐pro technology.

The surgeons were guided by the models in four specific phases of the procedure. For each of these phases, the surgeons had to rate, using 10‐point Likert scale questionnaires, the concordance of the virtual models with the anatomy of the intraoperative images and their usefulness in guiding the identification of the structures of interest.

## 3 STEP BY STEP 3D MODEL‐DIGITAL TWIN PRODUCTION

A contrast‐enhanced high‐resolution abdominal CT scan is obtained. Using dedicated software, images in DICOM format are processed by the bioengineer. The renal parenchyma is segmented through a process of selective thresholding, which involves distinguishing and categorizing voxels based on their grayscale values. The renal pedicle and the arteries supplying the tumour are reconstructed using the dynamic region growing technique. Subsequently, bioengineers and urologists collaboratively reviewed the virtual models to assess the accuracy of the segmentation. The final phase involved developing the mathematical HA3D model. A specific texture is produced for each of the virtual elements constituting, in the case of the kidney, by the parenchyma, the vessels, the tumour and the collecting system. The texture is depicted from a pool of intraoperative robotic surgery images using machine‐learning algorithms provided by dedicated software on the basis of the brightness and definition characteristics of each anatomical element. Once the textures are obtained, they are imported and assigned to each geometry of the model using software that adjusts their size and orient them on the three‐dimensional mesh.

**FIGURE 1:**
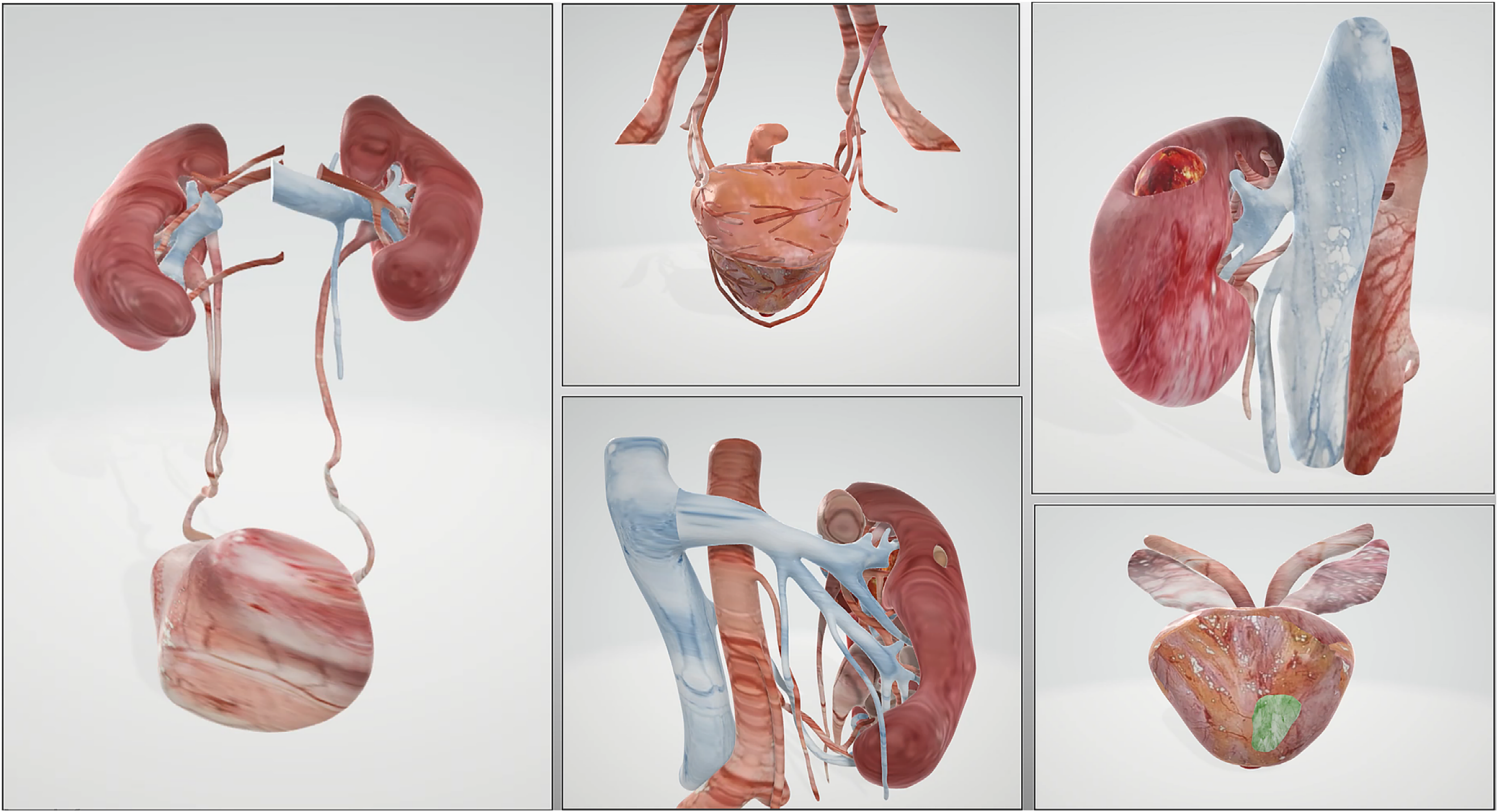
Image representing the final graphical rendering of 3D virtual digital‐twin models of various urological organs, built based on the textures of real organs acquired by the camera during the intraoperative phases of robotic procedures.

The final result is an interactive dynamic PDF 3D virtual model ‐ digital twin – reproducing not only the shape but also the appearance of the real organ. Depending on the organ of interest, different texture models are depicted, while each of them can be painted with a colour to highlight specific features (Figure 1). The production of a 3D model‐digital twin, from the DICOM image processing to the PDF building, takes approximately 45 minutes.

## 4 STEP BY STEP 3D MODEL‐DIGITAL TWIN‐GUIDED RAPN

**FIGURE 2:**
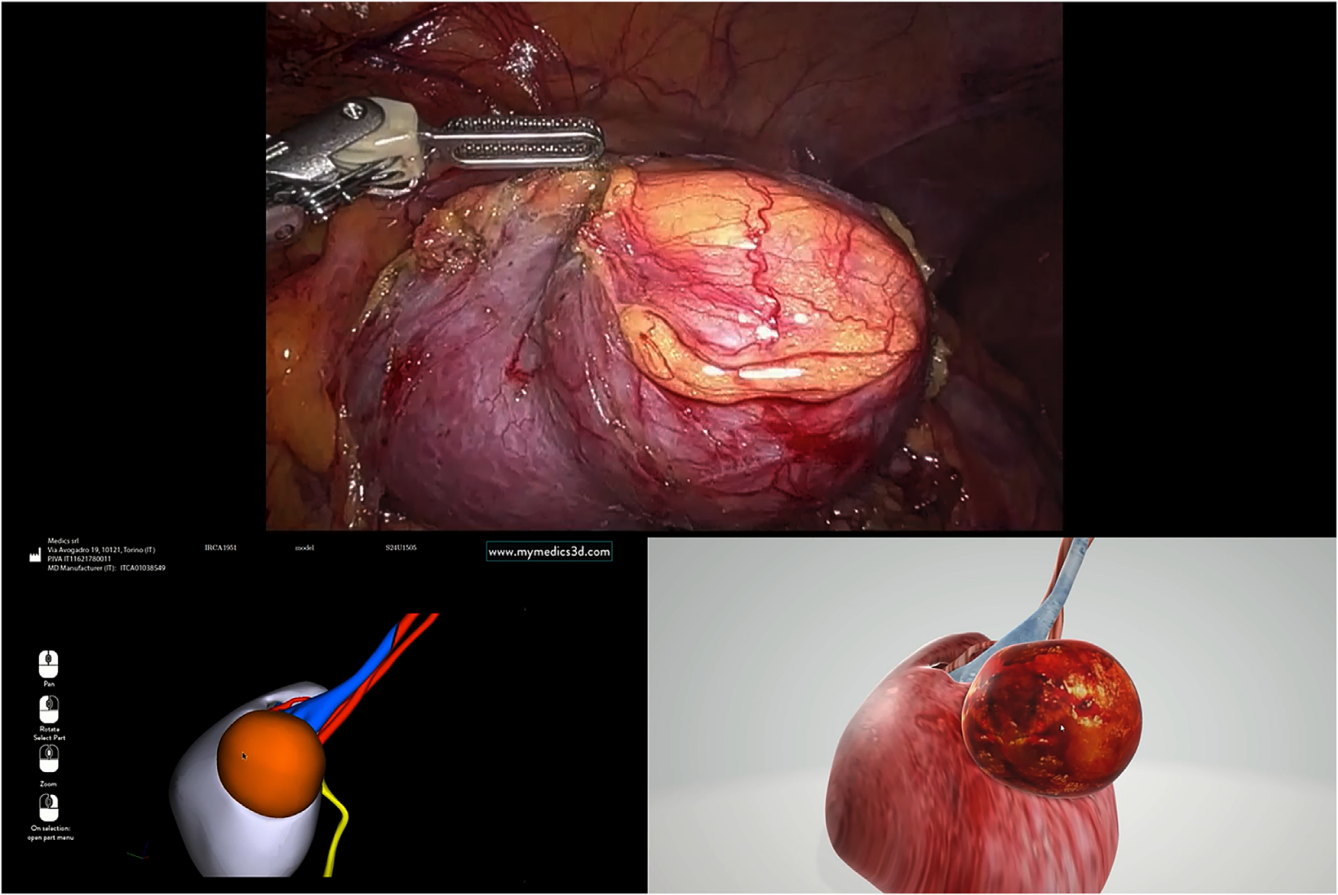
Intraoperative image demonstrating the difference between the guidance of an old‐generation 3D virtual model, lacking texture characteristics similar to real anatomy and a digital twin of the same organ, much more realistic.

A computer containing the dynamic PDF with the digital twin model is connected to the robotic console via the input port. The surgical procedure begins as usual, and the robot is docked. Access to the renal compartment is gained, and the kidney is isolated. At this stage, the TilePro mode of the robotic console software is activated, displaying the virtual model of the kidney in the adjunct screen under the intraoperative image during some specific steps of the RAPN procedure (Figure 2). The first step involves identifying the kidney and its boundaries. The second step consists of locating and dissecting the renal vascular pedicle. The third step focuses on identifying the renal tumour, and the fourth step involves locating the renal pelvis and ureter. During all these steps, the model can be manipulated via 3D mouse to display only specific components, hide others or change its orientation by rotating along its axes, according to the surgeon's instructions. After the steps are completed, the surgery proceeds according to the standard technique (See Video file attached).

## 5 RESULTS

Four patients, two per each surgeon harbouring low and intermediate complexity (PADUA 6 and 8) renal masses respectively, were selected for the study.

Both surgical procedures were guided by the aid of 3D images within the TilePro, with a projection time of 12.5 and 14.4 minutes. In both cases, it was possible to correctly identify the four anatomical structures of interest. The overall operative time was 92 and 104 minutes. No intraoperative or postoperative complications were recorded.

From the assessment made by the surgeons at the end of each procedure, the 3D digital twin models were found to be superior to their standard counterparts both in terms of perceived concordance with real anatomy and in usefulness to guide the identification of the tumour, vascular pedicle and ureter, while they did not demonstrate significant advantages in identifying the kidney and its margins (Table 1). Additionally, the young surgeon recorded a greater rate gap between the use of the 3D standard and the digital twin model for both the variables considered in each phase of the procedure.

**TABLE 1:** Evaluation of concordance of the virtual models with the anatomy of the intraoperative images and usefulness of the virtual models in identifying the real anatomical structures. For each of these surgical phases, the surgeons had to rate the two models, using 10‐point Likert scale questionnaire.

Experienced surgeon				Young surgeon			
**Concordance with anatomy**	Likert‐Scale			**Concordance with anatomy**	Likert‐Scale		
**PADUA 6**	**SURGICAL STEP**	**HA3DM**	**Digital twin**	**PADUA 6**	**SURGICAL STEP**	**HA3DM**	**Digital twin**
	Kidney identification	8/10	10/10		Kidney identification	8/10	10/10
	Pedicle identification	6/10	10/10		Pedicle identification	7/10	10/10
	Tumour identification	7/10	9/10		Tumour identification	8/10	10/10
	Ureter identification	6/10	8/10		Ureter identification	6/10	8/10
**PADUA 8**	**SURGICAL STEP**	**HA3DM**	**Digital twin**	**PADUA 8**	**SURGICAL STEP**	**HA3DM**	**Digital twin**
	Kidney identification	8/10	10/10		Kidney identification	9/10	10/10
	Pedicle identification	6/10	10/10		Pedicle identification	8/10	10/10
	Tumour identification	8/10	10/10		Tumour identification	9/10	10/10
	Ureter identification	7/10	8/10		Ureter identification	8/10	9/10
**Usefulness for navigation**		Likert‐Scale		**Usefulness for navigation**		Likert‐Scale	
**PADUA 6**	**SURGICAL STEP**	**HA3DM**	**Digital twin**	**PADUA 6**	**SURGICAL STEP**	**HA3DM**	**Digital twin**
	Kidney identification	7/10	7/10		Kidney identification	9/10	9/10
	Pedicle identification	8/10	9/10		Pedicle identification	8/10	10/10
	Tumour identification	7/10	8/10		Tumour identification	8/10	9/10
	Ureter identification	7/10	9/10		Ureter identification	8/10	9/10
**PADUA 8**	**SURGICAL STEP**	**HA3DM**	**Digital twin**	**PADUA 8**	**SURGICAL STEP**	**HA3DM**	**Digital twin**
	Kidney identification	8/10	10/10		Kidney identification	9/10	9/10
	Pedicle identification	8/10	9/10		Pedicle identification	8/10	10/10
	Tumour identification	8/10	9/10		Tumour identification	9/10	10/10
	Ureter identification	7/10	9/10		Ureter identification	8/10	9/10

## 6 LIMITS

Among the limitations of the present study, the subjective evaluation of 3D model‐digital twin does not allow for a definitive assessment of the utility and eventual superiority over the standard models. Further objective investigations, including an adequate population and statistical analyses, are mandatory to define their role in surgical practice.

Regarding the specific characteristics of the 3D model‐digital twin, an intrinsic limit may arise when evaluating the anatomical relationships between the tumour and renal parenchyma or vascular/urinary tract structures. In fact, in this case, the enhanced texture of the digital twin might not be effectively helpful in discriminating tumour contact surface compared to the standard models based on a conceptual colour‐code. For this reason, whenever needed, it is possible to rapidly switch to the standard fashion of the same virtual model to be guided during the tumour dissection and the resection bed suturing.

## 7 CONCLUSIONS

In conclusion, the new 3D digital twin models may represent a step forward towards the personalization of virtual reconstructions. Approaching real anatomy more closely while remaining digital tools, they offer the surgeon, especially a novice, a perceived higher degree of concordance with the intraoperative environment, making it easier to identify the structures of interest during the surgical procedure.

## AUTHOR CONTRIBUTIONS

*Manuscript writing*: Daniele Amparore and Alberto Piana. *Data collectio*n: Sabrina De Cillis and Federico Piramide. *Supervision*: Enrico Checcucci, Cristian Fiori and Francesco Porpiglia.

## CONFLICT OF INTEREST STATEMENT

All the authors have nothing to declare.

## Supporting information


**Video S1:** The video illustrates the rationale and production technology of the new generation of 3D models with anatomical textures called ‘digital twins’. Additionally, it demonstrates their application during the intraoperative phase of a robotic‐assisted partial nephrectomy, as well as the study's objective, methods and results.
